# Constitutional methylation of cancer-related and selenoprotein coding genes in breast carcinoma in Polish population

**DOI:** 10.1186/1897-4287-10-S4-A13

**Published:** 2012-12-10

**Authors:** Satish Gupta, Katarzyna Jaworska, Anna Jakubowska, Tomasz K Wojdacz, Lise Lotte Hansen, Jan Lubiński

**Affiliations:** 1 Department of Genetics and Pathology, Pomeranian Medical University, Szczecin, Poland; 2 Institute of Human Genetics, Aarhus University, Aarhus, Denmark; 3 Postgraduate School of Molecular Medicine, Warsaw Medical University, Poland

## 

Recently, much attention is paid to the phenomenon of gene’s hypermethylation in the peripheral blood (PB) and its involvement in the pathology of cancer. Breast cancer is a complex disease driven by multiple factors including both genetic and epigenetic processes. Genetic changes associated with breast cancer are not completely known, but epigenetic mechanisms involved in this disease seem to play an important role in its pathophysiology. An aberrant methylation in the promoter regions of genes involved in cancer induction and promotion, like *BRCA1*, *BRCA2*, *ATM*, *MLH1* and *ESR1*, may be of particular importance in breast cancer.

A very interesting class of genes, involved in selenium metabolism is under investigation with respect to different kind of cancers including breast cancer. Proteins coded by these genes, e.g. glutathione peroxidases, thioredoxin reductases or other selenoproteins are involved in variety of biological processes, ranging from DNA synthesis to protection against oxidative stress and may be related to breast cancer risk.

Aim of our study was to analyze methylation of genes involved in selenium metabolism (*GPX1*, *GPX4*, *TXNRD1*, *SEP15* and *SELT*) and other cancer related genes (*BRCA1*, *BRCA2*, *ATM*, *MLH1* and *ESR1*) in DNA isolated from PB of breast cancer patients or unaffected individuals.

A study was conducted on 30 female *BRCA1* mutation carriers, 30 female *CHEK2* mutation carriers and 36 unselected breast cancer patients negative for recurrent Polish *BRCA1*/*BRCA2* mutations and with specific pathological characteristics: medullary or atypical medullary breast cancer or bilateral breast cancer or ER, PGR and HER negative tumors. Methylation of *BRCA1* gene was additionally analyzed in group of 36 healthy controls, age-matched to unselected breast cancer patients. Methylation analysis was done by MS-HRM technique.

We observed promoter methylation of *BRCA1* (20 samples), *ESR1* (7 samples) and *MLH1* (3 samples) genes. *BRCA1* gene methylation was detected in 1 patient with *CHEK2* and 4 patients with *BRCA1* mutation. We found strong statistically significant difference of BRCA1 gene methylation in unselected breast cancer patients (15 patients) and unaffected age-matched controls (2 individuals): OR=12.1; p-value=0.0009. Selenoprotein coding genes *GPX1* and *GPX4* shows complete methylation while *TXNRD1* and *SELT* show methylation in ~10% of the studied group.

## Conclusion

Constitutional methylation (in peripheral blood) of *BRCA1* gene seems to be a strong factor risk of breast cancer with specific pathological characteristic.

